# Whole genome sequence of pan drug-resistant clinical isolate of *Acinetobacter baumannii* ST1890

**DOI:** 10.1371/journal.pone.0264374

**Published:** 2022-03-09

**Authors:** Thanwa Wongsuk, Siriphan Boonsilp, Anchalee Homkaew, Konrawee Thananon, Worrapoj Oonanant

**Affiliations:** 1 Department of Clinical Pathology, Faculty of Medicine Vajira Hospital, Navamindradhiraj University, Dusit, Bangkok, Thailand; 2 Division of Central Laboratory and Blood Bank, Faculty of Medicine Vajira Hospital, Navamindradhiraj University, Dusit, Bangkok, Thailand; 3 Research Facilitation Division, Faculty of Medicine Vajira Hospital, Navamindradhiraj University, Dusit, Bangkok, Thailand; 4 Research Unit in Integrative Immuno-Microbial Biochemistry and Bioresponsive Nanomaterials, Department of Microbiology, Faculty of Dentistry, Chulalongkorn University, Pathumwan, Bangkok, Thailand; 5 Department of Basic Medical Science, Faculty of Medicine Vajira Hospital, Navamindradhiraj University, Dusit, Bangkok, Thailand; Suez Canal University, EGYPT

## Abstract

*Acinetobacter baumannii* is an opportunistic gram-negative bacteria typically attributed to hospital-associated infection. It could also become multidrug-resistant (MDR), extensively drug-resistant (XDR), and pan drug-resistant (PDR) during a short period. Although *A*. *baumannii* has been documented extensively, complete knowledge on the antibiotic-resistant mechanisms and virulence factors responsible for pathogenesis has not been entirely elucidated. This study investigated the drug resistance pattern and characterized the genomic sequence by *de novo* assembly of PDR *A*. *baumannii* strain VJR422, which was isolated from a catheter-sputum specimen. The results showed that the VJR422 strain was resistant to any existing antibiotics. Based on *de novo* assembly, whole-genome sequences showed a total genome size of 3,924,675-bp. *In silico* and conventional MLST analysis of sequence type (ST) of this strain was new ST by Oxford MLST scheme and designated as ST1890. Moreover, we found 10,915 genes that could be classified into 45 categories by Gene Ontology (GO) analysis. There were 1,687 genes mapped to 34 Kyoto Encyclopedia of Genes and Genomes (KEGG) pathways. The statistics from Clusters of Orthologous Genes (COG) annotation identified 3,189 genes of the VJR422 strain. Regarding the existence of virulence factors, a total of 59 virulence factors were identified in the genome of the VJR422 strain by virulence factors of pathogenic bacteria databases (VFDB). The drug-resistant genes were investigated by searching in the Comprehensive Antibiotic Resistance Database (CARD). The strain harbored antibiotic-resistant genes responsible for aminoglycoside, β-lactam-ring-containing drugs, erythromycin, and streptogramin resistance. We also identified resistance-nodulation-cell division (RND) and the major facilitator superfamily (MFS) associated with the antibiotic efflux pump. Overall, this study focused on *A*. *baumannii* strain VJR422 at the genomic level data, i.e., GO, COG, and KEGG. The antibiotic-resistant genotype and phenotype as well as the presence of potential virulence associated factors were investigated.

## Introduction

*Acinetobacter baumannii* is an opportunistic, gram-negative coccobacilli [1] commonly associated with hospital-acquired nosocomial infections that can cause bacteremia, pneumonia, meningitis, and urinary tract infections [2, 3]. It is also considered the most common organism in the intensive care unit (ICU) and has been recognized as an emerging cause of nosocomial outbreaks globally [3]. The Infectious Diseases Society of America (IDSA) reported that *A*. *baumannii* ranked in the six top for priority dangerous microorganisms [4].

With serious concern for a multidrug-resistant (MDR) crisis, multidrug-resistant *A*. *baumannii* is one of the most alarming strains in terms of treatment and control. MDR has been increased all over the world that is considered a public health threat. Several recent investigations reported the emergence of multidrug-resistant bacterial pathogens from different origins including humans, birds, cattle, and fish that increase the need for routine application of the antimicrobial susceptibility testing to detect the antibiotic of choice as well as the screening of the emerging MDR strains [5–15]. MDR exhibited antibiotic resistance to different antibiotic including β-lactams, fluoroquinolones, tetracyclines, and aminoglycosides [16]. One of the major explanations for multidrug-resistant *A*. *baumannii* is the expression of resistance-nodulation-division (RND) transporters or outer membrane proteins, which actively pump drugs out of the cells [17, 18]. Treatment with colistin and tigecycline has become the only remaining active antibiotics treatment and the last resort in terms of treatment for MDR-*A*. *baumannii* [19–21]. Therefore, MDR-*A*. *baumannii* was also suggested as being extensively drug-resistant (XDR), which refers to resistance to all antibiotics except colistin and tigecycline, and an XDR-*A*. *baumannii* is a common cause of severe healthcare-associated infections worldwide [22, 23]. Recently, pan drug-resistant (PDR) *A*. *baumannii* strains have also been reported to resist colistin and tigecycline [24–27]. The emergence of PDR*-A*. *baumannii* has increased mortality rates and limited treatment management because monotherapy treatment is insufficient for curing [28–30]. Several virulence factors responsible for the pathogenicity of *A*. *baumannii* have been identified, including pilus, outer membrane porins, phospholipases, proteases, lipopolysaccharides, capsular polysaccharides, protein secretion systems, and iron-chelating systems. Some strains share genes related to adherence, invasion and survival, and form biofilms on the surface [31, 32].

*Acinetobacter baumannii* is one of the most successful pathogens responsible for hospital-acquired nosocomial infections because of the high prevalence of infections and scarcity of effective antibiotics for treatment. To overcome this problem, knowledge of the antibiotics-resistant mechanism and virulence factors responsible for pathogenesis is necessary.

Advances in whole-genome sequencing technology have facilitated bacterial whole-genome characterization, enhancing the ability to elucidate the antibiotic-resistant mechanism and pathogenesis in *Acinetobacter baumannii* [33, 34]. However, data concerning genome analysis on colistin resistance of *Acinetobacter baumannii* isolated from Thailand is currently limited in the literature [35]. To understand the antibiotics-resistant mechanism and virulence factors in *A*. *baumannii*, we described the whole genome of PDR*-A*. *baumannii* strain VJR422 by using *de novo* assembly with Illumina technology. The prediction of gene annotation and functional annotation employed a public database. Genome studies were also applied to predict potential antibiotics-resistant genes and virulence factors in this strain. The identification of genes involved in antibiotics resistance as well as virulence factors could be a potentially rewarding step towards a better understanding of the mechanism for antibiotics resistance in *A*. *baumannii* and could also provide foundational information for developing potential clinical management and treatment in the future.

## Materials and methods

### Ethics statement

This study was approved by the Ethics Committee of the Faculty of Medicine Vajira Hospital, Navamindradhiraj University, Bangkok, Thailand (COA 009/2561). Informed consent was waived because the study and the analysis used anonymous clinical data.

### Isolation and identification of *Acinetobacter baumannii*

In this study, we obtained *A*. *baumannii* strain VJR422 isolated from the single catheter-aspirated sputum of a patient who received care at our hospital in 2017, at the Faculty of Medicine Vajira Hospital, Navamindradhiraj University, Bangkok, Thailand. The bacterial strain was cultured on 5% sheep blood agar, chocolate agar, and MacConkey agar (commercially prepared by Clinical Diagnostics Ltd., Part, Bangkok, Thailand), followed by incubation for 18–24 hours at 35°C. Subsequently, the strain was identified using the matrix-assisted laser desorption/ionization time-of-flight (MALDI-TOF) (Bruker Microflex, Bremen, Germany).

### Antimicrobial susceptibility testing

Antimicrobial susceptibility testing was also performed by using a BD Phoenix NMIC-203 commercial kit (Becton Dickinson Diagnostic Systems, Sparks, Maryland, USA) on the BD Phoenix Automated Microbiology System (Becton Dickinson Diagnostic Systems, Spark, Maryland, USA). The manufacturer’s instructions were followed. Determining the minimum inhibitory concentration (MIC) breakpoint was done according to Clinical and Laboratory Standards Institute (CLSI) guidelines (M100, 27^th^ Ed.) [36]. The antimicrobials tested were cefoxitin, ceftazidime, ceftriaxone, imipenem, meropenem, aztreonam, ciprofloxacin, gentamicin, piperacillin/tazobactam, trimethoprim/sulfamethoxazole, tigecycline, and colistin. The control strains were *Escherichia coli* ATCC 25922 and *Pseudomonas aeruginosa* ATCC 27853 as recommended by the CLSI.

### Whole-genome DNA sequencing and analysis

Genomic DNA was extracted using a QIAamp DNA Mini Kit (QIAGEN, Hilden, Germany) following the manufacturer’s recommended protocol. The extracted DNA was visualized for quality on 0.8% w/v agarose gel electrophoresis, quantified with a NanoDrop 2000 spectrophotometer (Thermo Fisher, Wilmington, DE, USA), and stored at −30°C until further use. The poly-A-tailed DNA was ligated to paired-end adaptors and PCR amplified with a 500-bp insert. A mate-pair library was constructed with an insert size of 5 kb at Beijing Novogene Bioinformatics Technology Co., Ltd., Beijing, China. Whole-genome sequencing was performed on the Illumina platform with MPS (massively parallel sequencing) technology. Paired-end low-quality reads, mate-pair library, and PCR adaptor read were filtered by the quality control step using a Beijing Novogene Bioinformatics Technology pipeline. All good-quality paired reads were assembled using SOAP *de novo* (http://soap.genomics.org.cn/soapdenovo.html) into several scaffolds [37, 38]. In the next step, the filter reads were processed by gap closing.

### *In silico* Multilocus Sequence Typing (MLST)

*In silico* Multilocus Sequence Typing (MLST) and sequence types (STs) from whole-genome sequence data was performed using the MLST 2.0 (Software version: 2.0.1 (2018-08-15), Database version: 2.0.0 (2018-07-23) on the CGE server (http://www.genomicepidemiology.org) [39–45]. The Oxford and Pasteur MLST schemes for *A*. *baumannii* were tested. After the sequences of the predicted gene were uploaded, the allelic profile and STs were generated. The goeBURST diagram was constructed by Phyloviz software (http://www.phyloviz.net/goeburst/) [46].

### Conventional MLST

Genomic DNA was extracted by using the QIAamp DNA Mini Kit (QIAGEN, Hilden, Germany). MLST was carried out on extracted DNA using the methodology described by Bartual et al. In brief, fragments of seven housekeeping genes (*gltA*, *gyrB*, *gdhB*, *recA*, *cpn60*, *gpi*, and *rpoD*) were amplified by the polymerase chain reaction (PCR). The amplified PCR products were then purified using polyethylene glycol-sodium chloride (PEG-NaCl) precipitation (20% w/v of PEG, 2.5 M NaCl). Both strands of all PCR products were fully sequenced by A T G C Co; Ltd. (Pathum Thani, Thailand). The obtained sequences were assigned allele numbers by using the MLST website (https://pubmlst.org/organisms/acinetobacter-baumannii). The ST code was generated based on the combination of detected alleles for *gltA*, *gyrB*, *gdhB*, *recA*, *cpn60*, *gpi*, and *rpoD* with the Oxford scheme [40].

### Genome component prediction

The coding genes were predicted using GeneMarks [47]. The tandem repeats were predicted by the TRF (Tandem repeats finder) [48], and the repetitive sequences were analyzed by the RepeatMasker (http://www.repeatmasker.org/) [49]. The Transfer RNA (tRNA) and Ribosome RNA (rRNA) genes were predicted by the tRNAscan-SE [50] and rRNAmmer [51], respectively. Small nuclear RNAs (snRNA) were analyzed using BLAST against the Rfam database [52, 53].

### Functional gene annotation

Three databases were used to predict gene function: 1. GO—The Gene Ontology database [54], 2. KEGG—The Kyoto Encyclopedia of Genes and Genomes [55–57], and 3. COG—Protein sequences can be classified into Clusters of Orthologous Groups of proteins [58]

Virulence factors were analyzed using the VFDB (Virulence Factors of Pathogenic Bacteria) [59] for a virulence factor search. The abundance of resistant genes was detected by using the RGI program (version 4.2.2) to identify drug resistant genes by comparison with the reference genome in the Comprehensive Antibiotic Resistance Database (https://card.mcmaster.ca/) (submitted file to the online database and analyzed on 4^th^ January 2020).

### Expression levels of efflux pump genes

Total RNA was extracted using conventional hot phenol RNA extraction and converted into cDNA using a cDNA synthesis kit (iScript Reverse Transcription Supermix, Bio-Rad, Hercules, CA, United States). The quality and purity of the RNA were evaluated using a Nanodrop-100 spectrophotometer (Nanodrop Technology Inc., Wilmington, DE, USA). Real-time quantification of cDNA was carried out on a CFX96 Touch TM real-time PCR detection system (Biorad, California, USA) using the iScript SYBR green PCR master mix (Bio-Rad, Hercules, CA, United States). The amplification cycle included initial denaturation at 95°C for 1 minute and 40 cycles of denaturation at 95°C for 15 seconds followed by annealing and extension at 62°C for 30 seconds. The primers used for amplification included *ade*B_RT_F: GGATTATGGCGACTGAAGGA and *ade*B_RT_R: AATACTGCCGCCAATACCAG for *ade*B [60], *ade*G_RT_F: CGTAACTATGCGGTGCTCAA and *ade*G_RT_R: ATCGCGTAGTCACCAGAACC for *ade*G [60], and *ade*J_RT_F: CATCGGCTGAAACAGTTGAA and *ade*J_RT_R: GCCTGACCATTACCAGCACT for *ade*J [60]. Relative expression values were determined using the 2 –^ΔΔCt^ method. *A*. *baumannii* ATCC 19606 was used as a standard strain for normalization of relative mRNA levels.

## Results and discussion

### Bacterial isolate, identification, and antimicrobial susceptibility test

*A*. *baumannii* strain VJR422 was isolated from the catheter-aspirated sputum of a patient who admitted to Vajira hospital. Colonies on blood agar and chocolate agar were smooth, raised, and opaque with non-lactose fermenter colonies on MacConkey agar. The antimicrobial susceptibility profile of this strain was resistant to all antibiotics tested, as shown in Table 1.

**Table 1:** Antibiotic sensitivity of *A*. *baumannii* strain VJR422.

Drugs	Concentration (μg/ml)
Cefoxitin	>16
Ceftazidime	> 16
Ceftriaxone	>16
Imipenem	>8
Meropenem	>8
Aztreonam	>16
Ciprofloxacin	> 2
Gentamicin,	>8
Piperacillin/Tazobactam	>64/4
Trimethoprim/Sulfamethoxazole	>2/38
Tigecycline	>4
Colistin	>4

### Multilocus sequence typing

**Fig 1:**
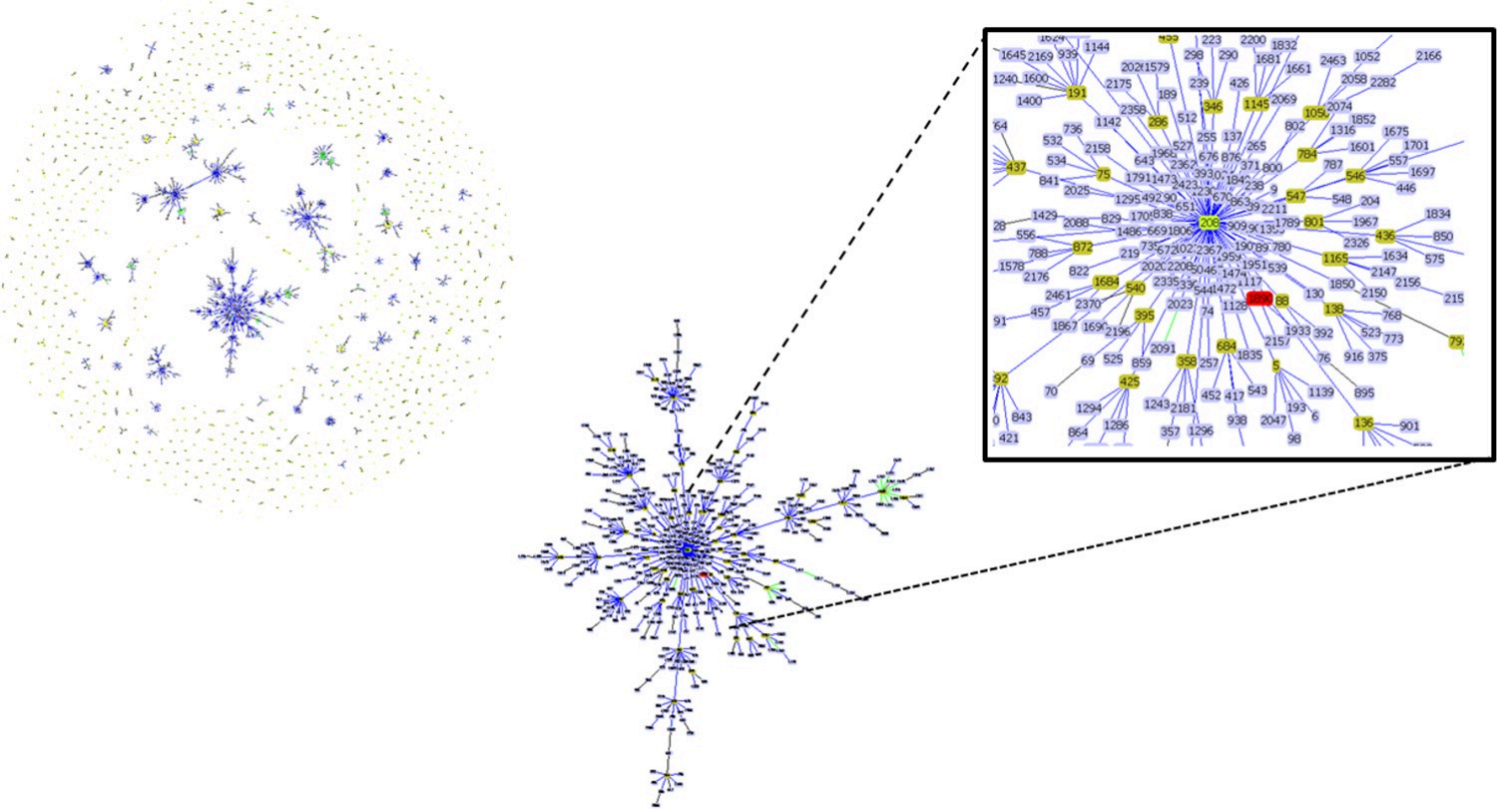
Genetic population structure of *A*. *baumannii* obtained by goeBURST analysis using the 2,479 ST currently deposited in the MLST database. Two STs are linked when they differ in just one of seven loci (SLV analysis). Single STs represent singletons. goeBURST clonal cluster CC208 containing ST1890 (in red) found in this study. The putative founding and subgroup genotype are indicated in green and yellow, respectively.

Firstly, we performed MLST bioinformatics analyses from whole genome sequence on the CGE server. The sequence type of *A*. *baumannii* VJR422 was ST2 (2-2-2-2-2-2-2) based on the seven housekeeping genes in the Pasteur MLST scheme (*cpn*60, *fus*A, *glt*A, *pyr*G, *rec*A, *rpl*B, and *rpo*B) [61], which belong to international Clone II (IC-II) [62]. Interestingly, the sequence type of *A*. *baumannii* VJR422 was new ST (1-3-3-2-2-202-3), which represents a new combination of existing alleles based on the seven housekeeping genes of the Oxford MLST scheme (*glt*A, *gyr*B, *gdh*B, *rec*A, *cpn*60, *gpi*, and *rpo*D) [40, 63]. Therefore, we confirmed this new ST by performing wet-lab analyses of conventional MLST (Oxford MLST scheme). This new ST was submitted to the *A*. *baumannii* MLST (Oxford) database at PubMLST.org: Public databases for molecular typing (https://pubmlst.org/) and designated as ST1890 (Sender—Dr. Worrapoj Oonanant; Profile added by a curator—Dr. Paul Higgins—on February 20, 2019; 12.05). MLST data can be represented in groups and clonal complexes (CCs), including evolutionary descent patters by the goeBurst. As shown in Fig 1, we found that the new ST1890, which is the single locus variant (SLV) of ST208 (1-3-3-2-2-97-3), differs in its *gpi* loci and belongs to clonal complex 208 (CC208), which has also been reported in South Korea and India [64–66]. Therefore, these STs might have emerged successively by variations in *gpi* loci.

### Functional annotation of the genomic sequence of *A*. *baumannii* VJR422

**Fig 2:**
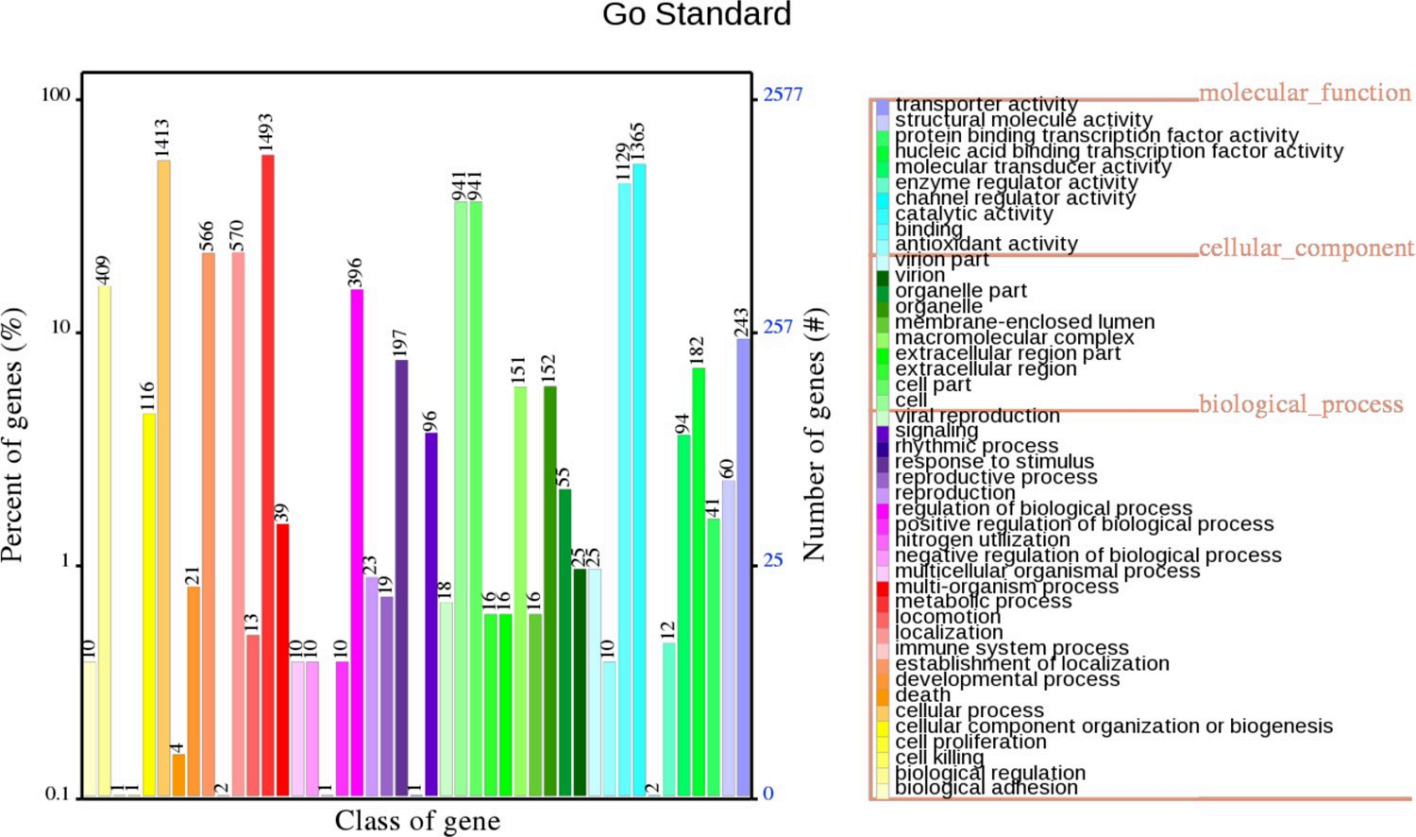
GO annotation of VJR422. The horizontal axis displays the GO function class for the annotated genes. The right vertical axis is the gene number, and the left vertical axis is the percentage of gene number annotated in all the coding genes.

After *de novo* assembly, the complete genome of VJR422 was 3,924,675 bp with a GC content of 40.01%. The general characteristics of the genome are summarized in Table 2. In the GO annotation results, the gene functions could be detected, and the statistics of GO annotation are listed in Fig 2. In the GO analysis, 10,915 genes were classified into 45 categories including 1) molecular function (10 categories), which are catalytic activity (1,365 genes), binding (1,129 genes), transporter activity (243 genes), nucleic acid binding transcription factor activity (182 genes), molecular transducer activity (94 genes), structural molecule activity (60 genes), protein binding transcription factor activity (41 genes), enzyme regulator activity (12 genes), antioxidant activity (10 genes), and channel regulator activity (2 genes), and 2) Cellular component (10 categories), which are cell part (941 genes), cell (941 genes), organelle (152 genes), macromolecule complex (151 genes), organelle part (55 genes), virion part (25 genes), virion (25 genes), membrane-enclosed lumen (16 genes), extracellular region part (16 genes), and extracellular region (16 genes), and 3) biological process (25 categories), which include the cellular process (1,493 genes), metabolic process (1,413 genes), localization (570 genes), the establishment of localization (566 genes), biological regulation (409 genes), regulation of biological process (396 genes), response to stimulus (197 genes), cellular component organization or biogenesis (116 genes), viral reproduction (18 genes), signaling (96 genes), multi-organism process (39 genes), reproductive process (19 genes), reproduction (23 genes), developmental process (21 genes), locomotion (13 genes), positive regulation of biological process (10 genes), nitrogen utilization (10 genes), biological adhesion (10 genes), multicellular organismal process (10 genes), death (4 genes), immune system process (2 genes), negative regulation of biological process (1 gene), cell proliferation (1 gene), cell killing (1 gene), and rhythmic process (1 gene). The predominance of each part is shown in S2 Table.

**Table 2:** Assemble and annotation of *A*. *baumannii* VJR422.

Descriptions	
Length of genome sequence (bp)	3,924,675
Annotated total gene number	3,730
Annotated total gene length (bp)	3,439,146
% GC content in gene sequence	40.01
% Gene length to genome length	87.63
Gene average length (bp)	922
Gene internal length (bp)	485,529
% Gene internal GC content	31.2
% Gene internal length to genome length	12.37
Number of tRNA	61
Number of sRNA	1
Number of 5s (*de novo*)	6
Number of 16s (*de novo*)	0
Number of 23s (*de novo*)	0

**Fig 3:**
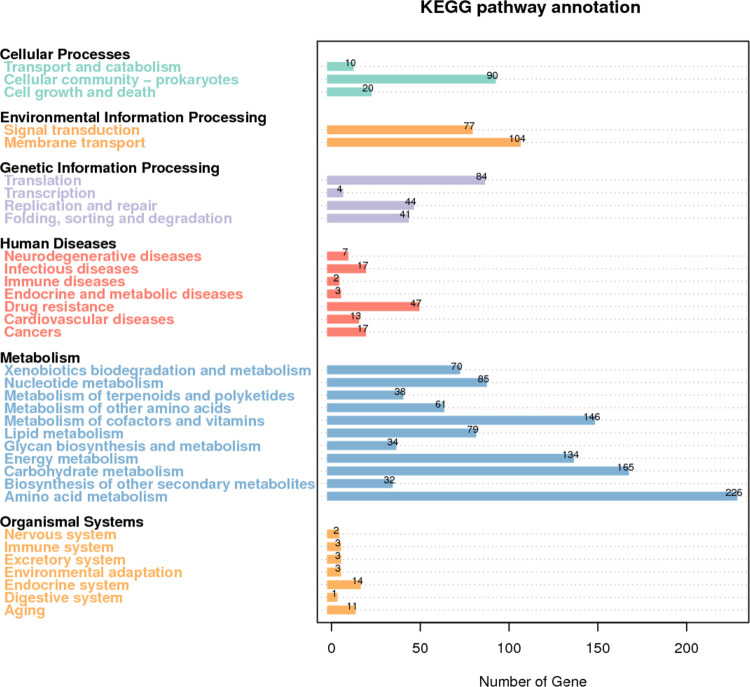
KEGG annotation of VJR422. The horizontal axis is KEGG pathway type, and the vertical axis is the number of annotated genes.

For analysis by the KEGG database to the related annotated gene of VJR422, a total of 1,687 genes were mapped to 35 KEGG pathways. The metabolism group comprised 1,070 genes, representing significantly more coding genes than other pathways. Those are associated with amino acid metabolism (226 genes), carbohydrate metabolism (165 genes), metabolism of cofactors and vitamins (146 genes), energy metabolism (134 genes), nucleotide metabolism (85 genes), lipid metabolism (79 genes), xenobiotic degradation and metabolism (70 genes), metabolism of other amino acids (61 genes), metabolism of terpenoids and polyketides (38 genes), glycan biosynthesis and metabolism (34 genes), and biosynthesis of other secondary metabolites (32 genes) (Fig 3 and S2 Table). In the cellular process group, there are 120 of the genes linked to cellular community-prokaryote (90 genes), cell growth and death (20 genes), as well as transport and catabolism (10 genes). The environmental information-processing group included 181 genes (104 genes of membrane and transport pathways and 77 genes of signal transduction pathways). Besides, 173 genes linked to the genetic information processing group, including translation (84 genes), replication and repair (44 genes), folding, sorting, and degradation (41 genes), and transcription (4 genes). Meanwhile, 106 genes linked to human diseases, including drug resistance (47 genes), infectious diseases (17 genes), cancers (17 genes), cardiovascular disease (13 genes), neurodegenerative diseases (7 genes), endocrine and metabolic diseases (3 genes), and immune diseases (2 genes). Finally, 31 genes linked to the organismal system, including endocrine system (14 genes), aging (11 genes), immune system (3 genes), excretory system (3 genes), environmental adaptation (3 genes), nervous system (2 genes), and digestive system (1 gene).

**Fig 4:**
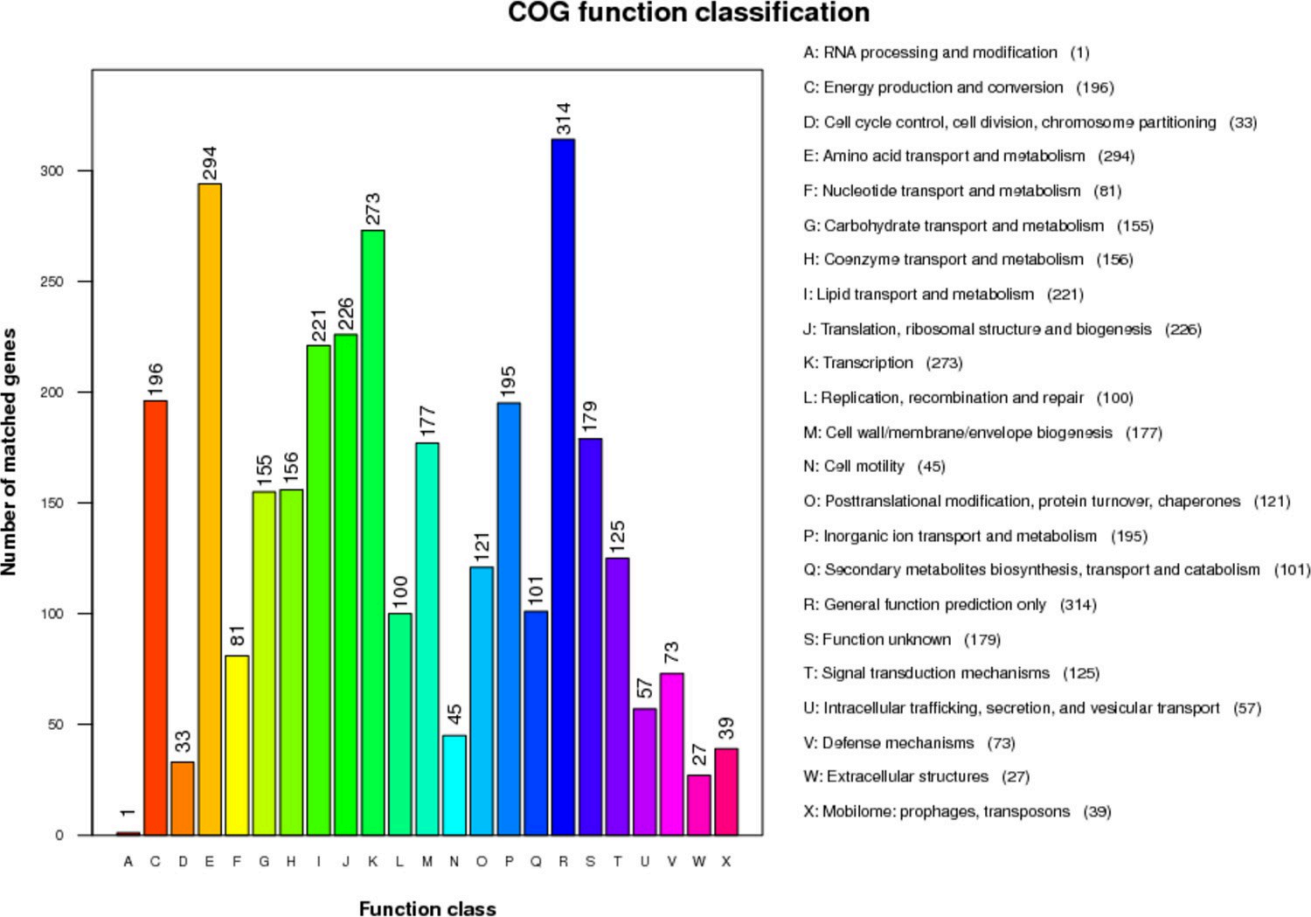
COG annotation of VJR422. The horizontal axis is COG function type, and the vertical axis is the number of annotated genes.

The COG database is divided into 25 parts according to function. The results in Fig 4. were obtained from the statistics of COG annotation for the VJR422 strain. A total of 3,189 genes were annotated and classified into 22 functional groups. No genes were allocated to the chromatin structure and dynamics, nuclear structure, and cytoskeleton functional domains. Among the COG functional classifications, “General function prediction only” comprised the largest group (314 genes), followed by “Amino acid transport and metabolism” (294 genes), and “Transcription” (273 genes). Moreover, 179 genes were classified as “Function unknown” (Fig 4.).

The COG categories in the genome of VJR422 were divided into four main domains comprising 1) Information storage and processing, 2) Cellular process and signaling, 3) Metabolism, and 4) Poorly characterized [62, 67]. All were compared with another COG toward the genome of *A*. *baumannii* DMS06669 [68] and *A*. *baumannii* XH386 [69] in Table 3. The predominance of VJR422 includes extracellular structures, annotated genes, secondary metabolite biosynthesis, transport, and catabolism-annotated genes.

**Table 3:** Comparison of COG among the genome of *A*. *baumanni* strain VJR422, DMS06669, and XH386.

Category	Class	Functional description	Strains		
			VJR422	DMS06669	XH386
Information storage and processing	B	Chromatin structure and dynamics	0	0	0
	L	Replication, recombination and repair	100	101	131
	K	Transcription	273	268	269
	A	RNA processing and modification	1	2	1
	J	Translation, ribosomal structure and biogenesis	226	210	235
Cellular process and signaling	O	Posttranslational modification, protein turnover, chaperones	121	124	121
	U	Intracellular trafficking, secretion, and vesicular transport	57	56	55
	W	Extracellular structures	27	3	3
	Z	Cytosleleton	0	0	0
	N	Cell motility	45	14	55
	M	Cell wall/membrane/envelope biogenesis	177	164	186
	T	Signal transduction mechanisms	125	81	117
	V	Defense mechanisms	73	74	66
	Y	Nuclear structure	0	0	0
	D	Cell cycle control, cell division, chromosome partitioning	33	30	39
Metabolism	Q	Secondary metabolites biosynthesis, transport and catabolism	101	62	67
	P	Inorganic ion transport and metabolism	195	188	183
	I	Lipid transport and metabolism	221	169	221
	H	Coenzyme transport and metabolism	156	99	143
	F	Nucleotide transport and metabolism	81	70	82
	C	Energy production and conversion	196	177	201
	G	Carbohydrate transport and metabolism	155	109	153
	E	Amino acid transport and metabolism	294	263	263
Poorly characterized	X	Mobilome: prophages, transposons	39	41	N
	S	Function unknown	179	193	219
	R	General function prediction only	314	211	238

### Virulence factors and drug-resistance genes of *A*. *baumannii* VJR422

A total of 59 virulence factors were identified in the genome of *A*. *baumannii* VJR422, including adherence (outer membrane protein), biofilm formation (*Ade*FGH efflux pump/transport autoinducer, biofilm-associated protein, Csu pili, and polysaccharide poly-n-acetylglucosamine), enzyme (phospholipase C and phospholipase D), immune evasion (LPS and capsule), iron uptake (acinetobactin and heme utilization), regulation (quorum sensing and two-component system), serum resistance (*Pbp*G), and stress adaptation (catalase) (S3 Table).

**Fig 5:**
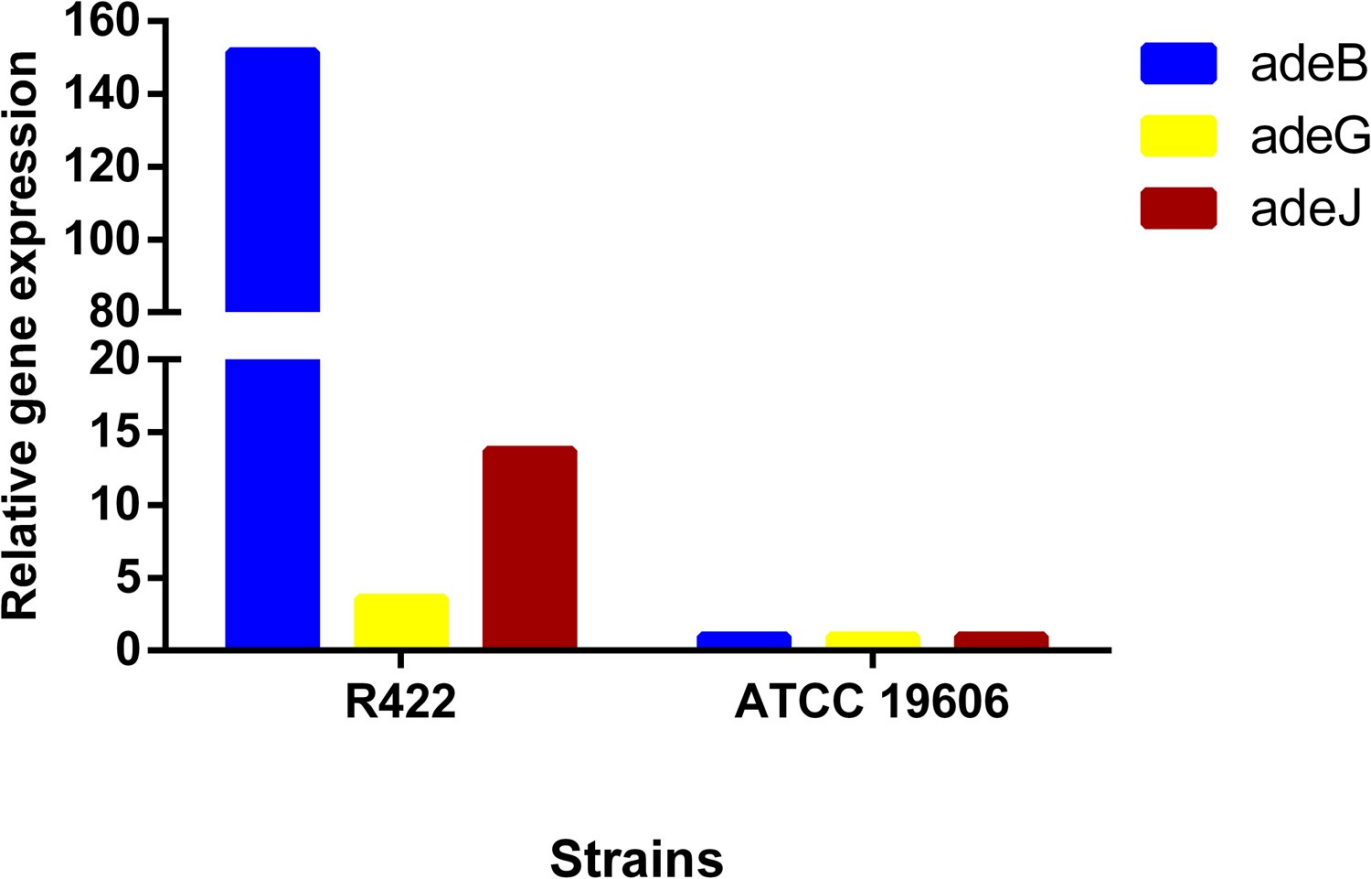
The gene expression level of *ade*B, *ade*G, and *ade*J in clinical isolates *A*. *baumannii* strain VJR422 increased 151.51, 3.57, and 13.76 times respectively compared with susceptible strain ATCC19606.

The abundance of resistant genes in the VJR422 strain was assessed by searching the Comprehensive Antibiotic Resistance Database (CARD). The CARD includes BLAST and the Resistance Gene Identifier (RGI) software tools for the analysis of molecular sequences, the prediction of the resistome based on homology and single nucleotide polymorphism (SNP) models. The distribution of antibiotic resistance genes in the genome of VJR422 is also shown in S4 Table. A total of 25 genes that respond to different mechanisms of drug-resistance in *Acinetobacter* were identified by the CARD tool. The percentage identity of the matching region with the reference sequence in the program was in a range from 98.98% to 100%. *A*. *baumannii* VJR422 was resistant to aminoglycosides (gentamycin and ciprofloxacin), and genes responsible for aminoglycoside resistance (*arm*A, APH(6)-Id, APH(3’’)-Ib, and ANT(3’’)-IIc) were found. The 16S rRNA methylase, which confers high-level resistance on all aminoglycosides encoded by the *arm*A gene, was initially identified in *Citrobacter freundii* in Poland in 2002 and has now been identified worldwide among gram-negative bacteria [70–73]. Commonly, resistance to aminoglycoside is conferred by aminoglycoside-modifying enzymes (acetyltransferases, nucleotidyltransferases, and phosphotransferases) [74]. The VJR422 was found to possess variants of phosphotransferases, i.e. APH(6)-Id and APH(3’’)-Ib, and ANT(3’’)-IIc, a variant of nucleotidyltransferase. Four β-lactamase-encoding genes that can hydrolyze antimicrobials containing a β-lactam ring were predicted, i.e. TEM-1, ADC-73, OXA-23, and OXA-66. The VJR422 strain was resistant to all β-lactam-ring-containing drugs (ceftazidime, imipenem, meropenem, cefoxitin, ceftriaxone, and aztreonam.) TEM-1 (Temoneira-1) β-lactamase is one of the best-known drug-resistant enzymes able to hydrolyze penicillin and the first generation of cephalosporin [75]. The ADC-73 (*Acinetobacter*-derived cephalosporinase-73) β-lactamase is regarded as a chromosomally encoded class C β-lactamase that confers resistance to penicillin, cephalosporin, and β-lactam/β-lactamase inhibitor combinations [76]. Therefore, the VJR422 strain is also resistant to piperacillin/tazobactam. The class D carbapenem-hydrolyzing oxacillinases (OXA type), OXA-23 and OXA-66 were predicted in WGS of VJR422. OXA-23 is one of the largest groups of OXA-type carbapenemases in *A*. *baumannii*, and OXA-66 is a variant of OXA-51 classified as an OXA-51-like group of enzymes [77, 78]. Regarding the Ade pump, resistance-nodulation-cell division (RND) transporter genes (*ade*ABC, *ade*FGK, *ade*N, and *ade*RS) were identified in the VJR422 genome analysis. Investigation of the tigecycline resistance mechanism in the VJR422 isolate was challenging. Several point mutations in the regulatory gene *ade*RS were observed, resulting in overexpression of the AdeABC efflux pump system. AdeIJK and AdeFGH showed high expression compared with the susceptible strain (ATCC 19606) (Fig 5). The presence of numbers and polyspecificities of RND transporters correlate with high intrinsic and clinical resistance in gram-negative bacilli [79]. Moreover, *Acinetobacter baumannii Aba*F, *Aba*Q, and *Amv*A, the major facilitator superfamily (MFS) antibiotic efflux pump, were also identified in the VJR422 genome. MFS transporters are involved in drug efflux systems and lead to antibiotic resistance in both gram-positive and gram-negative bacteria [80]. *Aba*F was identified as an efflux pump associated with fosfomycin resistance in *A*. *baumannii* [81, 82]. *Aba*Q is mainly involved in the extrusion of quinolone-type drugs in *A*. *baumannii* [83]. *AmvA* contributes resistance to erythromycin, acriflavine, benzalkonium chloride, and methyl viologen [81, 84]. Multidrug efflux pumps of the small multidrug resistance (SMR) type are made of a transport protein located in the inner membrane. *AbeS*, an efflux pump of the SMR type, was identified in the VJR422 genome. *AbeS* could decrease susceptibility in the various class of antibiotics, disinfectants, dyes, and detergents [85]. The *mph*E and *msr*E genes associated with erythromycin resistance and streptogramin resistance were identified as well [68, 86, 87].

## Conclusions

In conclusion, this study identified and characterized the MDR *A*. *baumannii* strain VJR422 from a clinical specimen using the WGS analysis tool at the Faculty of Medicine Vajira Hospital, Navamindradhiraj University. Knowledge of this bacterial pathogen at the genomic level has not been reported previously at our hospital. We reported and updated the new ST1890 in the PubMlst Database and characterized the VJR422 in the genomic level data, i.e. GO, COG, and KEGG. The antibiotic resistance genotype and phenotype as well as the presence of potential virulence associated factors were investigated.

## Supporting information

S1 Table GO classification into three main parts in the genome of VJR422. (DOCX)

S2 Table KEGG classification into six main parts in the genome of VJR422. (DOCX)

S3 Table Virulence gene predicted by the VFDB (Virulence Factors of Pathogenic Bacteria). (DOCX)

S4 Table The distribution of antibiotic resistance genes in the genome of VJR422. (DOCX)
