# Supplementary material for: Whole genome sequence of pan drug-resistant clinical isolate of Acinetobacter baumannii ST1890
Source: PLoS One. 2022 Mar 9;17(3):e0264374. doi: 10.1371/journal.pone.0264374 (PMC8906637; doi:10.1371/journal.pone.0264374)
Supplement: S1 Table — (DOCX) [file pone.0264374.s001.docx]

**S1 Table.** GO classification into three main parts in the genome of VJR422

| **GO main parts** | **Categories** | **Annotated unigenes** | |
| --- | --- | --- | --- |
| **Molecular function** | Catalytic activity | 1,365 |  |
|  | Binding | 1,129 |  |
|  | Transporter activity | 243 |  |
|  | Nucleic acid binding transcription factor activity | 182 |  |
|  | Protein binding transcription factor activity | 41 |  |
|  | Molecular transducer activity | 94 |  |
|  | Structural molecule activity | 60 |  |
|  | Enzyme regulator activity | 12 |  |
|  | Antioxidant activity | 10 |  |
|  | Channel regulator activity | 2 |  |
| Cellular component | Cell | 941 |  |
|  | Cell part | 941 |  |
|  | Organelle | 152 |  |
|  | Macromolecule complex | 151 |  |
|  | Organelle part | 55 |  |
|  | Virion | 25 |  |
|  | Virion part | 25 |  |
|  | Extracellular region | 16 |  |
|  | Extracellular region part | 16 |  |
|  | Membrane-enclosed lumen | 16 |  |
| Biological process | Metabolic process | 1,493 |  |
|  | Mellular process | 1,413 |  |
|  | Localization | 570 |  |
|  | Establishment of localization | 566 |  |
|  | Biological regulation | 409 |  |
|  | Regulation of biological process | 396 |  |
|  | Response to stimulus | 197 |  |
|  | Cellular component organization or biogenesis | 116 |  |
|  | Signaling | 96 |  |
|  | Multi-organism process | 39 |  |
|  | Reproduction | 23 |  |
|  | Developmental process | 21 |  |
|  | Reproductive process | 19 |  |
|  | Viral reproduction | 18 |  |
|  | Locomotion | 13 |  |
|  | Biological adhesion | 10 |  |
|  | Nitrogen utilization | 10 |  |
|  | Positive regulaton of biological process | 10 |  |
|  | Multicellular organismal process | 10 |  |
|  | Death | 4 |  |
|  | Immune system process | 2 |  |
|  | Negative regulation of biological process | 1 |  |
|  | Cell proliferation | 1 |  |
|  | Cell killing | 1 |  |
|  | Rhythmic process | 1 |  |
