# Supplementary material for: Whole genome sequence of pan drug-resistant clinical isolate of Acinetobacter baumannii ST1890
Source: PLoS One. 2022 Mar 9;17(3):e0264374. doi: 10.1371/journal.pone.0264374 (PMC8906637; doi:10.1371/journal.pone.0264374)
Supplement: S2 Table — (DOCX) [file pone.0264374.s002.docx]

**S2 Table.** KEGG classification into six main parts in the genome of VJR422

| **KEGG** | **Categories** | **Annotated unigenes** |
| --- | --- | --- |
| Metabolism | Amino acid metabolism | 226 |
|  | Carbohydrate metabolism | 165 |
|  | Metabolism of cofactors and vitamins | 146 |
|  | Energy metabolism | 134 |
|  | Nucleotide metabolism | 85 |
|  | Lipid metabolism | 79 |
|  | Xenobiotics degradation and metabolism | 70 |
|  | Metabolism of other amino acids | 61 |
|  | Metabolism of terpenoides and polyketides | 38 |
|  | Glycan biosynthesis and metabolism | 34 |
|  | Biosynthesis of other secondary metabolites | 32 |
| Cellular process | Cellular community-prokaryote | 90 |
|  | Cell growth and death | 20 |
|  | Transport and catabolism | 10 |
| Environmental information processing | Membrane and transport pathway | 104 |
|  | Signal transduction pathway | 77 |
| Genetic information processing | Translation | 84 |
|  | Replication and repair | 44 |
|  | Folding, sorting and degradation | 41 |
|  | Transcription | 4 |
| Human diseses | Drug resistance | 47 |
|  | Infectious diseases | 17 |
|  | Cancers | 17 |
|  | Cardiovascular disease | 13 |
|  | Neurodegenerative diseases | 7 |
|  | Endocrine and metabolic diseases | 3 |
|  | Immune diseases | 2 |
| Organismal system | Endocrine system | 14 |
|  | Aging | 11 |
|  | Immune system | 3 |
|  | Excretory system | 3 |
|  | Environmental adaptation | 3 |
|  | Nervous system | 2 |
|  | Digestive system | 1 |
