# Supplementary material for: Whole genome sequence of pan drug-resistant clinical isolate of Acinetobacter baumannii ST1890
Source: PLoS One. 2022 Mar 9;17(3):e0264374. doi: 10.1371/journal.pone.0264374 (PMC8906637; doi:10.1371/journal.pone.0264374)
Supplement: S3 Table — (DOCX) [file pone.0264374.s003.docx]

**S3 Table.** Virulence gene predicted by the VFDB (Virulence Factors of Pathogenic Bacteria)

| **Virulence** | **Relatated gene** | **Predicted in VJR422** |
| --- | --- | --- |
| **Adherence (1 Items)** |  |  |
| Outer membrane protein | *omp*A | R422_GM002237 |
| **Biofilm formation (14 Items)** |  |  |
| AdeFGH efflux pump/transport auto inducer | *ade*F | R422_GM001745 |
| AdeFGH efflux pump/transport auto inducer | *ade*G | R422_GM001746 |
| AdeFGH efflux pump/transport auto inducer | *ade*H | R422_GM001747 |
| Biofilm-associated protein | *bap* | R422_GM002250 |
| Csu pili | *csu*A/B | R422_GM003494 |
| Csu pili | *csu*A | R422_GM003493 |
| Csu pili | *csu*B | R422_GM003492 |
| Csu pili | *csu*C | R422_GM003491 |
| Csu pili | *csu*D | R422_GM003490 |
| Csu pili | *csu*E | R422_GM003489 |
| PNAG (Polysaccharide poly-N-acetylglucosamine) | *pga*A | R422_GM001958 |
| PNAG (Polysaccharide poly-N-acetylglucosamine) | *pga*B | R422_GM001959 |
| PNAG (Polysaccharide poly-N-acetylglucosamine) | *pga*C | R422_GM001960 |
| PNAG (Polysaccharide poly-N-acetylglucosamine) | *pga*D | R422_GM001961 |
| **Enzyme (2 Items)** |  |  |
| [Phospholipase C](about:blank) | *plc*C | R422_GM003033 R422_GM003294 |
| [Phospholipase D](about:blank) | *plcD* | R422_GM003506 |
| **Immune evasion (8 Items)** |  |  |
| LPS | *lps*B | R422_GM000822 |
| LPS | *lpx*A | R422_GM001233 |
| LPS | *lpx*B | R422_GM000343 |
| LPS | *lpx*C | R422_GM001185 |
| LPS | *lpx*D | R422_GM001231 |
| LPS | *lpx*L | R422_GM000823 |
| LPS | *lpx*M | R422_GM000614 |
| Capsule | -- | R422_GM003004  to R422_GM003026 |
| **Iron uptake (28 Items)** |  |  |
| Acinetobactin | *bar*A | R422_GM002416 |
| Acinetobactin | *bar*B | R422_GM002417 |
| Acinetobactin | *bas*A | R422_GM002402 |
| Acinetobactin | *bas*B | R422_GM002403 |
| Acinetobactin | *bas*C | R422_GM002410 |
| Acinetobactin | *bas*D | R422_GM002411 |
| Acinetobactin | *bas*F | R422_GM002413 |
| Acinetobactin | *bas*G | R422_GM002414 |
| **Virulence** | **Relatated gene** | **Predicted in STR422** |
| Acinetobactin | *bas*H | R422_GM002418 |
| Acinetobactin | *bas*I | R422_GM002419 |
| Acinetobactin | *bas*J | R422_GM002420 |
| Acinetobactin | *bau*A | R422_GM002409 |
| Acinetobactin | *bau*B | R422_GM002408 |
| Acinetobactin | *bau*C | R422_GM002406 |
| Acinetobactin | *bau*D | R422_GM002405 |
| Acinetobactin | *bau*E | R422_GM002407 |
| Acinetobactin | *bau*F | R422_GM002401 |
| Acinetobactin | *ent*E | R422_GM002412 |
| Heme utilization | *hem*O | R422_GM000959 |
| Heme utilization | -- | R422_GM000966 |
| Heme utilization | -- | R422_GM000965 |
| Heme utilization | -- | R422_GM000964 |
| Heme utilization | -- | R422_GM000963 |
| Heme utilization | -- | R422_GM000962 |
| Heme utilization | -- | R422_GM000961 |
| Heme utilization | -- | R422_GM000960 |
| Heme utilization | -- | R422_GM000958 |
| Heme utilization | -- | R422_GM000957 |
| **Regulation (4 Items)** |  |  |
| Quorum sensing | *aba*I | R422_GM002488 |
| Quorum sensing | *aba*R | R422_GM002486 |
| Two-component system | *bfm*R | R422_GM001836 |
| Two-component system | *bfm*S | R422_GM001835 |
| **Serum resistance (1 Item)** |  |  |
| PbpG | *pbp*G | R422_GM003192 |
| **Stress adaptation (1 Item**) |  |  |
| Catalase (*Neisseria*) | *kat*A | R422_GM002784 |
