# Supplementary material for: Whole genome sequence of pan drug-resistant clinical isolate of Acinetobacter baumannii ST1890
Source: PLoS One. 2022 Mar 9;17(3):e0264374. doi: 10.1371/journal.pone.0264374 (PMC8906637; doi:10.1371/journal.pone.0264374)
Supplement: S4 Table — (DOCX) [file pone.0264374.s004.docx]

**S4 Table.** The distribution of antibiotic resistance genes in the genome of VJR422

| **RGI** | **Antimicrobial resistance gene family** | **Gene** | **Resistance mechanism** | **% identity of matching region** | **% length of reference sequence** | **Predicted in VJR422** |
| --- | --- | --- | --- | --- | --- | --- |
| Perfect | 16S rRNA methyltransferase (G1405) | armA | antibiotic target alteration | 100.0 | 100.00 | R422_GM003475_1 |
| Strict | APH(6) | APH(6)-Id | antibiotic inactivation | 99.64 | 100.00 | R422_GM003624_1 |
| Strict | APH(3'') | APH(3'')-Ib | antibiotic inactivation | 99.25 | 105.24 | R422_GM003625_1 |
| Strict | ANT(3'') | ANT(3'')-IIc | antibiotic inactivation | 70.16 | 101.16 | R422_GM002466_1 |
| Perfect | TEM beta-lactamase | TEM-1 | antibiotic inactivation | 100.0 | 100.00 | R422_GM003726_1 |
| Perfect | ADC beta-lactamase without carbapenemase activity | ADC-73 | antibiotic inactivation | 100.0 | 100.00 | R422_GM002426_1 |
| Perfect | OXA beta-lactamase | OXA-66 | antibiotic inactivation | 100.0 | 100.00 | R422_GM002122_1 |
| Perfect | OXA beta-lactamase | OXA-23 | antibiotic inactivation | 100.0 | 100.00 | R422_GM003724_1 |
| Perfect | resistance-nodulation-cell division (RND) antibiotic efflux pump | adeL | antibiotic efflux | 100.0 | 100.00 | R422_GM001743_1 |
| Perfect | resistance-nodulation-cell division (RND) antibiotic efflux pump | adeG | antibiotic efflux | 100.0 | 100.00 | R422_GM001745_1 |
| Strict | resistance-nodulation-cell division (RND) antibiotic efflux pump | adeF | antibiotic efflux | 99.81 | 100.00 | R422_GM001380_1 |
| Perfect | resistance-nodulation-cell division (RND) antibiotic efflux pump | adeC | antibiotic efflux | 100.0 | 100.00 | R422_GM002607_1 |
| Perfect | resistance-nodulation-cell division (RND) antibiotic efflux pump | adeB | antibiotic efflux | 100.0 | 100.00 | R422_GM002608_1 |
| Strict | resistance-nodulation-cell division (RND) antibiotic efflux pump | adeR | antibiotic efflux | 99.19 | 100.00 | R422_GM002610_1 |
| Perfect | resistance-nodulation-cell division (RND) antibiotic efflux pump | adeK | antibiotic efflux | 100.0 | 100.00 | R422_GM002292_1 |
| Strict | resistance-nodulation-cell division (RND) antibiotic efflux pump | adeN | antibiotic efflux | 99.08 | 100.00 | R422_GM001219_1 |
| Perfect | resistance-nodulation-cell division (RND) antibiotic efflux pump | adeA | antibiotic efflux | 100.0 | 100.00 | R422_GM002609_1 |
| Strict | major facilitator superfamily (MFS) antibiotic efflux pump | *Acinetobacter baumannii* AbaQ | antibiotic efflux | 99.76 | 94.47 | R422_GM001367_1 |
| Perfect | major facilitator superfamily (MFS) antibiotic efflux pump | Acinetobacter baumannii AbaF | antibiotic efflux | 100.0 | 100.00 | R422_GM001746_1 |
| Strict | major facilitator superfamily (MFS) antibiotic efflux pump | Acinetobacter baumannii AmvA | antibiotic efflux | 98.98 | 100.00 | R422_GM003292_1 |
| Perfect | small multidrug resistance (SMR) antibiotic efflux pump | abeS | antibiotic efflux | 100.0 | 100.00 | R422_GM001738_1 |
| Strict | major facilitator superfamily (MFS) antibiotic efflux pump | tetR | antibiotic target alteration, antibiotic efflux | 100.0 | 99.52 | R422_GM003619_1 |
| Strict | major facilitator superfamily (MFS) antibiotic efflux pump | tet(B) | antibiotic efflux | 99.5 | 101.0 | R422_GM003618_1 |
| Perfect | ABC-F ATP-binding cassette ribosomal protection protein | msrE | antibiotic target protection | 100.0 | 100.00 | R422_GM003472_1 |
| Perfect | macrolide phosphotransferase (MPH) | mphE | antibiotic inactivation | 100.0 | 100.00 | R422_GM003471_1 |
